# Eucomic acid methanol monosolvate

**DOI:** 10.1107/S1600536811030017

**Published:** 2011-07-30

**Authors:** Guo-Qiang Li, Yao-Lan Li, Guo-Cai Wang, Zhi-Hong Liang, Ren-Wang Jiang

**Affiliations:** a Guangdong Province Key Laboratory of Pharmacodynamic Constituents of Traditional Chinese Medicine and New Drugs Research, Institute of Traditional Chinese Medicine and Natural Products, Jinan University, Guangzhou 510632, People’s Republic of China; b Analysis and Testing Center, Jinan University, Guangzhou 510632, People’s Republic of China

## Abstract

In the crystal structure of the title compound [systematic name: 2-hy­droxy-2-(4-hy­droxy­benz­yl)butane­dioic acid methanol monosolvate], C_11_H_12_O_6_·CH_3_OH, the dihedral angles between the planes of the carboxyl groups and the benzene ring are 51.23 (9) and 87.97 (9)°. Inter­molecular O—H⋯O hydrogen-bonding inter­actions involving the hy­droxy and carb­oxy­lic acid groups and the methanol solvent mol­ecule give a three-dimensional structure.

## Related literature

For general background to natural existance and related structures, see: Jiang *et al.* (2006 ▶); Li *et al.* (2008 ▶). For the absolute configuration of eucomic acid, see: Heller & Tamm (1974 ▶).
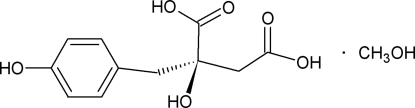


## Experimental

### 

#### Crystal data

C_11_H_12_O_6_·CH_4_O
*M* *_r_* = 272.25
Orthorhombic, 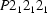

*a* = 5.8970 (2) Å
*b* = 7.2088 (3) Å
*c* = 31.3290 (4) Å
*V* = 1331.81 (7) Å^3^
*Z* = 4
Mo *K*α radiation
μ = 0.11 mm^−1^
*T* = 293 K
0.60 × 0.20 × 0.10 mm

#### Data collection

Bruker SMART 1000 CCD diffractometer
7417 measured reflections
1408 independent reflections
1184 reflections with *I* > 2σ(*I*)
*R*_int_ = 0.036

#### Refinement

*R*[*F* ^2^ > 2σ(*F* ^2^)] = 0.030
*wR*(*F* ^2^) = 0.081
*S* = 1.05
1408 reflections
178 parameters
H-atom parameters constrained
Δρ_max_ = 0.18 e Å^−3^
Δρ_min_ = −0.19 e Å^−3^

### 

Data collection: *SMART* (Bruker, 1998 ▶); cell refinement: *SMART* and *SAINT* (Bruker, 1998 ▶); data reduction: *XPREP* in *SHELXTL* (Sheldrick, 2008 ▶); program(s) used to solve structure: *SHELXS97* (Sheldrick, 2008 ▶); program(s) used to refine structure: *SHELXL97* (Sheldrick, 2008 ▶); molecular graphics: *OLEX2* (Dolomanov *et al.*, 2009 ▶); software used to prepare material for publication: *OLEX2*.

## Supplementary Material

Crystal structure: contains datablock(s) I, global. DOI: 10.1107/S1600536811030017/zs2127sup1.cif

Structure factors: contains datablock(s) I. DOI: 10.1107/S1600536811030017/zs2127Isup2.hkl

Supplementary material file. DOI: 10.1107/S1600536811030017/zs2127Isup3.cml

Additional supplementary materials:  crystallographic information; 3D view; checkCIF report

## Figures and Tables

**Table 1:** Hydrogen-bond geometry (Å, °)

*D*—H⋯*A*	*D*—H	H⋯*A*	*D*⋯*A*	*D*—H⋯*A*
O1—H1⋯O3^i^	0.82	1.97	2.781 (3)	170
O2—H2⋯O1^ii^	0.82	2.33	2.861 (2)	123
O4—H4⋯O2^iii^	0.82	1.85	2.639 (2)	162
O6—H6⋯O7	0.82	1.76	2.575 (4)	170
O7—H7⋯O5^iv^	0.82	1.93	2.694 (4)	156

Symmetry codes: (i) 
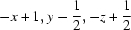
; (ii) 
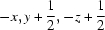
; (iii) 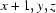; (iv) 
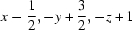
.

**Fig. 1.**
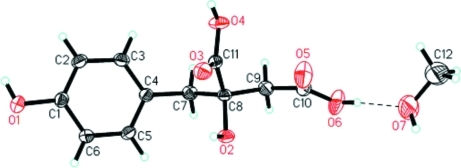
The molecular structure of the title compound showing 30% probability displacement ellipsoids and the atom-numbering scheme. The intermolecular hydrogen bond is shown as a dashed line.

## supplementary crystallographic information

### Comment

The title compound C_11_H_12_O_6_.CH_3_OH (Fig. 1) is the monomethanol solvate of eucomic acid [systematic name: 2-hydroxy-2-(4-hydroxybenzyl)butanedioic acid], and was originally isolated from the stems of *Opuntia dillenii* (Jiang *et al.*, 2006) and the absolute configuration was established by synthesis (Heller & Tamm, 1974). With the present compound,which was isolated from the stems of the related species *Opuntia vulgaris*, the dihedral angle between the plane of the benzene ring and that of the carboxylic group at C8 is 51.23 (9)°, and 87.97 (9)° with that at C9. These values are similar to those in the methyl eucomate structure (Li *et al.*, 2008).

Intermolecular O—H···O hydrogen-bonding interactions involving the hydroxy and carboxylic acid groups and the methanol molecule (Table 1) give a three-dimensional structure. A short intramolecular interaction between the C8 hydroxy group and a carboxyl O acceptor is also present [O2—H2···O3 = 2.655 (2) Å; <O—H···O = 117°].

### Experimental

The title compound was isolated from the stems of Opuntia vulgaris, 1 kg of which was extracted with 95% ethanol at room temperature, then concentrated by rotary evaporator. The crude extract was suspended in distilled water and partitioned with petroleum ether, ethyl acetate and n-butanol. The title compound (22 mg) was isolated from the n-butanol fraction using silica-gel column chromatography. Crystals of the title compound were obtained after slow evaporation of a methanolic solution at room temperature.

### Refinement

The C-bound H atoms were positioned geometrically and were included in the refinement in the riding-model approximation, with C—H = 0.96 Å (CH_3_) and *U*_iso_(H) = 1.5*U*_eq_(C); 0.97 Å (CH_2_) and *U*_iso_(H) = 1.2*U*_eq_(C); 0.93 Å (aryl H) and *U*_iso_(H)= 1.2*U*_eq_(C); O—H = 0.82 Å and *U*_iso_(H) = 1.5*U*_eq_(O). The absolute configuration determined by Heller & Tamm (1974) by analysis was invoked, having (for the numbering scheme used in this determination) C8(*R*). Friedel pairs in the data set (934) were merged.

### Crystal data

**Table:**

C_11_H_12_O_6_·CH_4_O	*D*_x_ = 1.358 Mg m^−^^3^
*M**_r_* = 272.25	Mo *K*α radiation, λ = 0.71073 Å
Orthorhombic, *P*2_1_2_1_2_1_	Cell parameters from 2341 reflections
*a* = 5.8970 (2) Å	θ = 1.3–25.0°
*b* = 7.2088 (3) Å	µ = 0.11 mm^−^^1^
*c* = 31.3290 (4) Å	*T* = 293 K
*V* = 1331.81 (7) Å^3^	Prism, colourless
*Z* = 4	0.60 × 0.20 × 0.10 mm
*F*(000) = 576	

### Data collection

**Table:**

Bruker SMART 1000 CCD diffractometer	1184 reflections with *I* > 2σ(*I*)
Radiation source: fine-focus sealed tube	*R*_int_ = 0.036
graphite	θ_max_ = 25.0°, θ_min_ = 1.3°
ω scans	*h* = −7→6
7417 measured reflections	*k* = −8→6
1408 independent reflections	*l* = −36→37

### Refinement

**Table:**

Refinement on *F*^2^	Primary atom site location: structure-invariant direct methods
Least-squares matrix: full	Secondary atom site location: difference Fourier map
*R*[*F*^2^ > 2σ(*F*^2^)] = 0.030	Hydrogen site location: inferred from neighbouring sites
*wR*(*F*^2^) = 0.081	H-atom parameters constrained
*S* = 1.05	*w* = 1/[σ^2^(*F*_o_^2^) + (0.0459*P*)^2^ + 0.0811*P*] where *P* = (*F*_o_^2^ + 2*F*_c_^2^)/3
1408 reflections	(Δ/σ)_max_ < 0.001
178 parameters	Δρ_max_ = 0.18 e Å^−^^3^
0 restraints	Δρ_min_ = −0.19 e Å^−^^3^

### Special details

**Table:**

Geometry. All e.s.d.'s (except the e.s.d. in the dihedral angle between two l.s. planes) are estimated using the full covariance matrix. The cell e.s.d.'s are taken into account individually in the estimation of e.s.d.'s in distances, angles and torsion angles; correlations between e.s.d.'s in cell parameters are only used when they are defined by crystal symmetry. An approximate (isotropic) treatment of cell e.s.d.'s is used for estimating e.s.d.'s involving l.s. planes.
Refinement. Refinement of *F*^2^ against ALL reflections. The weighted *R*-factor *wR* and goodness of fit *S* are based on *F*^2^, conventional *R*-factors *R* are based on *F*, with *F* set to zero for negative *F*^2^. The threshold expression of *F*^2^ > σ(*F*^2^) is used only for calculating *R*-factors(gt) *etc*. and is not relevant to the choice of reflections for refinement. *R*-factors based on *F*^2^ are statistically about twice as large as those based on *F*, and *R*-factors based on ALL data will be even larger.

### Fractional atomic coordinates and isotropic or equivalent isotropic displacement parameters (Å^2^)

**Table:**

	*x*	*y*	*z*	*U*_iso_*/*U*_eq_	
O4	0.5195 (3)	0.2588 (3)	0.38870 (6)	0.0496 (5)	
H4	0.6358	0.3081	0.3799	0.074*	
O2	−0.0623 (2)	0.3712 (2)	0.37354 (5)	0.0399 (4)	
H2	−0.0175	0.4478	0.3561	0.060*	
O1	0.2583 (3)	0.1398 (3)	0.18267 (5)	0.0503 (5)	
H1	0.3811	0.0962	0.1756	0.075*	
O3	0.3496 (3)	0.4842 (3)	0.35125 (5)	0.0465 (5)	
C5	−0.0136 (4)	0.1541 (3)	0.28608 (7)	0.0390 (6)	
H5	−0.1536	0.1885	0.2972	0.047*	
C4	0.1514 (4)	0.0859 (3)	0.31338 (7)	0.0346 (5)	
C1	0.2305 (4)	0.1201 (3)	0.22599 (7)	0.0373 (5)	
C8	0.1204 (4)	0.2530 (3)	0.38545 (7)	0.0337 (5)	
C3	0.3574 (4)	0.0333 (3)	0.29570 (7)	0.0401 (6)	
H3	0.4705	−0.0139	0.3133	0.048*	
O6	−0.0015 (5)	0.3883 (3)	0.49413 (6)	0.0750 (6)	
H6	0.0124	0.4873	0.5068	0.112*	
C6	0.0240 (4)	0.1722 (3)	0.24286 (7)	0.0380 (6)	
H6A	−0.0889	0.2193	0.2252	0.046*	
O5	0.2358 (4)	0.5185 (3)	0.44936 (6)	0.0769 (7)	
C11	0.3419 (4)	0.3486 (4)	0.37382 (7)	0.0361 (5)	
C2	0.3976 (4)	0.0497 (4)	0.25230 (7)	0.0420 (6)	
H2A	0.5365	0.0136	0.2409	0.050*	
C7	0.1074 (4)	0.0688 (3)	0.36073 (7)	0.0397 (6)	
H7A	0.2170	−0.0167	0.3728	0.048*	
H7B	−0.0421	0.0156	0.3649	0.048*	
C10	0.1187 (4)	0.3916 (4)	0.45945 (7)	0.0449 (6)	
C9	0.0974 (4)	0.2185 (4)	0.43323 (7)	0.0412 (6)	
H9A	−0.0491	0.1622	0.4388	0.049*	
H9B	0.2134	0.1311	0.4421	0.049*	
O7	0.0058 (6)	0.6834 (4)	0.54040 (10)	0.1137 (10)	
H7	−0.0761	0.7730	0.5357	0.171*	
C12	0.1744 (7)	0.7337 (6)	0.56770 (13)	0.1048 (14)	
H12A	0.3167	0.6874	0.5573	0.157*	
H12B	0.1446	0.6828	0.5954	0.157*	
H12C	0.1808	0.8666	0.5696	0.157*	

### Atomic displacement parameters (Å^2^)

**Table:**

	*U*^11^	*U*^22^	*U*^33^	*U*^12^	*U*^13^	*U*^23^
O4	0.0228 (8)	0.0617 (12)	0.0644 (12)	−0.0021 (9)	−0.0012 (8)	0.0092 (9)
O2	0.0262 (8)	0.0491 (10)	0.0446 (9)	−0.0002 (8)	0.0008 (7)	0.0065 (8)
O1	0.0462 (10)	0.0669 (13)	0.0378 (9)	0.0088 (10)	0.0061 (8)	−0.0085 (9)
O3	0.0389 (10)	0.0530 (11)	0.0474 (10)	−0.0084 (9)	0.0064 (8)	0.0099 (9)
C5	0.0297 (12)	0.0451 (14)	0.0421 (13)	0.0043 (12)	0.0037 (10)	−0.0067 (11)
C4	0.0293 (11)	0.0371 (12)	0.0374 (12)	−0.0031 (11)	−0.0008 (10)	−0.0057 (10)
C1	0.0362 (12)	0.0398 (13)	0.0360 (12)	−0.0020 (11)	0.0037 (10)	−0.0075 (11)
C8	0.0232 (11)	0.0434 (13)	0.0345 (12)	0.0004 (11)	−0.0002 (9)	0.0034 (10)
C3	0.0312 (13)	0.0442 (14)	0.0449 (13)	0.0002 (12)	−0.0058 (11)	−0.0028 (11)
O6	0.0911 (16)	0.0758 (14)	0.0580 (12)	−0.0167 (15)	0.0377 (12)	−0.0163 (10)
C6	0.0336 (13)	0.0430 (13)	0.0375 (13)	0.0072 (12)	−0.0018 (10)	−0.0022 (10)
O5	0.0892 (16)	0.0762 (15)	0.0653 (12)	−0.0429 (13)	0.0272 (12)	−0.0222 (11)
C11	0.0272 (12)	0.0485 (15)	0.0325 (12)	−0.0041 (12)	0.0018 (10)	−0.0049 (12)
C2	0.0287 (12)	0.0488 (14)	0.0485 (14)	0.0006 (12)	0.0033 (11)	−0.0114 (12)
C7	0.0344 (13)	0.0416 (13)	0.0430 (13)	−0.0068 (12)	0.0000 (10)	0.0015 (11)
C10	0.0399 (14)	0.0588 (16)	0.0361 (13)	−0.0049 (14)	0.0047 (11)	0.0011 (12)
C9	0.0361 (13)	0.0501 (14)	0.0373 (13)	−0.0072 (12)	0.0027 (10)	0.0033 (11)
O7	0.134 (2)	0.0946 (19)	0.112 (2)	0.0550 (19)	−0.042 (2)	−0.0518 (17)
C12	0.101 (3)	0.096 (3)	0.118 (3)	0.032 (3)	−0.028 (3)	−0.024 (3)

### Geometric parameters (Å, °)

**Table:**

O4—H4	0.8200	C3—H3	0.9300
O4—C11	1.317 (3)	C3—C2	1.385 (3)
O2—H2	0.8200	O6—H6	0.8200
O2—C8	1.424 (3)	O6—C10	1.298 (3)
O1—H1	0.8200	C6—H6A	0.9300
O1—C1	1.374 (3)	O5—C10	1.189 (3)
O3—C11	1.207 (3)	C2—H2A	0.9300
C5—H5	0.9300	C7—H7A	0.9700
C5—C4	1.386 (3)	C7—H7B	0.9700
C5—C6	1.378 (3)	C10—C9	1.499 (4)
C4—C3	1.388 (3)	C9—H9A	0.9700
C4—C7	1.511 (3)	C9—H9B	0.9700
C1—C6	1.380 (3)	O7—H7	0.8200
C1—C2	1.381 (3)	O7—C12	1.361 (4)
C8—C11	1.521 (3)	C12—H12A	0.9600
C8—C7	1.539 (3)	C12—H12B	0.9600
C8—C9	1.523 (3)	C12—H12C	0.9600
			
C11—O4—H4	109.5	O3—C11—C8	122.6 (2)
C8—O2—H2	109.5	C1—C2—C3	119.7 (2)
C1—O1—H1	109.5	C1—C2—H2A	120.2
C4—C5—H5	119.1	C3—C2—H2A	120.2
C6—C5—H5	119.1	C4—C7—C8	114.53 (19)
C6—C5—C4	121.8 (2)	C4—C7—H7A	108.6
C5—C4—C3	117.7 (2)	C4—C7—H7B	108.6
C5—C4—C7	120.9 (2)	C8—C7—H7A	108.6
C3—C4—C7	121.3 (2)	C8—C7—H7B	108.6
O1—C1—C6	117.1 (2)	H7A—C7—H7B	107.6
O1—C1—C2	122.8 (2)	O6—C10—C9	113.4 (2)
C6—C1—C2	120.1 (2)	O5—C10—O6	123.6 (2)
O2—C8—C11	108.42 (17)	O5—C10—C9	122.9 (2)
O2—C8—C7	110.29 (18)	C8—C9—H9A	108.9
O2—C8—C9	106.74 (18)	C8—C9—H9B	108.9
C11—C8—C7	108.24 (18)	C10—C9—C8	113.2 (2)
C11—C8—C9	112.71 (18)	C10—C9—H9A	108.9
C9—C8—C7	110.41 (19)	C10—C9—H9B	108.9
C4—C3—H3	119.4	H9A—C9—H9B	107.7
C2—C3—C4	121.2 (2)	C12—O7—H7	109.5
C2—C3—H3	119.4	O7—C12—H12A	109.5
C10—O6—H6	109.5	O7—C12—H12B	109.5
C5—C6—C1	119.5 (2)	O7—C12—H12C	109.5
C5—C6—H6A	120.2	H12A—C12—H12B	109.5
C1—C6—H6A	120.2	H12A—C12—H12C	109.5
O4—C11—C8	112.08 (19)	H12B—C12—H12C	109.5
O3—C11—O4	125.1 (2)		
			
O2—C8—C11—O4	171.94 (19)	C6—C5—C4—C7	179.4 (2)
O2—C8—C11—O3	−12.4 (3)	C6—C1—C2—C3	−0.5 (4)
O2—C8—C7—C4	68.0 (2)	O5—C10—C9—C8	−32.1 (4)
O2—C8—C9—C10	−62.6 (2)	C11—C8—C7—C4	−50.5 (3)
O1—C1—C6—C5	179.5 (2)	C11—C8—C9—C10	56.3 (3)
O1—C1—C2—C3	−179.8 (2)	C2—C1—C6—C5	0.2 (4)
C5—C4—C3—C2	0.6 (3)	C7—C4—C3—C2	−179.7 (2)
C5—C4—C7—C8	−77.1 (3)	C7—C8—C11—O4	−68.4 (2)
C4—C5—C6—C1	0.5 (4)	C7—C8—C11—O3	107.2 (2)
C4—C3—C2—C1	0.1 (4)	C7—C8—C9—C10	177.5 (2)
C3—C4—C7—C8	103.2 (3)	C9—C8—C11—O4	54.0 (3)
O6—C10—C9—C8	148.3 (2)	C9—C8—C11—O3	−130.3 (2)
C6—C5—C4—C3	−0.9 (3)	C9—C8—C7—C4	−174.29 (19)

### Hydrogen-bond geometry (Å, °)

**Table:**

*D*—H···*A*	*D*—H	H···*A*	*D*···*A*	*D*—H···*A*
O1—H1···O3^i^	0.82	1.97	2.781 (3)	170
O2—H2···O3	0.82	2.19	2.655 (2)	116
O2—H2···O1^ii^	0.82	2.33	2.861 (2)	123
O4—H4···O2^iii^	0.82	1.85	2.639 (2)	162
O6—H6···O7	0.82	1.76	2.575 (4)	170
O7—H7···O5^iv^	0.82	1.93	2.694 (4)	156

Symmetry codes: (i) −*x*+1, *y*−1/2, −*z*+1/2; (ii) −*x*, *y*+1/2, −*z*+1/2; (iii) *x*+1, *y*, *z*; (iv) *x*−1/2, −*y*+3/2, −*z*+1.
